# Pulmonary cryptococcosis mimicking neoplasm in terms of uptake PET/CT

**DOI:** 10.1590/0100-3984.2016.0121

**Published:** 2018

**Authors:** Lucas de Pádua Gomes de Farias, Igor Gomes Padilha, Márcia Rosana Leite Lemos, Carla Jotta Justo dos Santos, Christiana Maria Nobre Rocha de Miranda

**Affiliations:** 1 Universidade Federal de Alagoas (UFAL), Maceió, AL, Brazil; 2 Clínica de Medicina Nuclear e Radiologia de Maceió (MedRadius), Maceió, AL, Brazil

**Figure 1:**
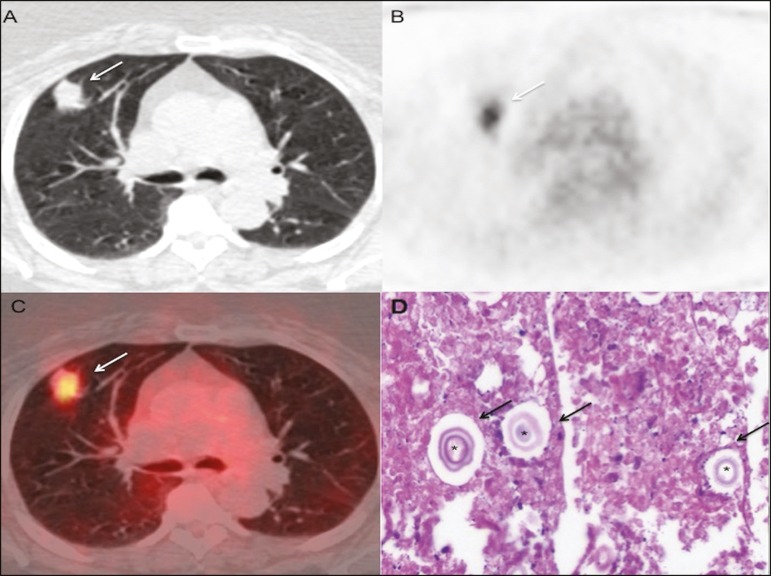
**A-C:** PET/CT scans showing aspiculated mass with soft tissue attenuation, located in the anterior segment of the right upper lobe, arising from the horizontal fissure and extending to the pleura (arrows), with a maximum SUV of 5.5. **D:** Histological slide, with hematoxylin-eosin staining (magnification, ×10), of a lung biopsy sample, showing rounded structures with basophilic capsules (asterisks), accompanied by nuclear fragmentation of inflammatory cells, together with macrophages and ultinucleated giant cells (arrows). Histochemical analysis, with mucicarmine and Grocott's staining, confirmed the presence of cryptococci.

Dear Editor,

We report the case of a 64-year-old female nonsmoker who presented with complaints of chronic cough and weight loss. Physical examination revealed no fever or other abnormalities. Positron emission tomography/computed tomography (PET/CT) demonstrated a mass, with soft tissue attenuation and spiculated borders, in the anterior segment of the right upper lobe, arising from the horizontal fissure and extending to the pleura, measuring 3.2 × 2.2 × 1.2 cm, with a maximum standardized uptake value (SUV) of 5.5 and high glycolytic metabolism (Figure 1A-C). The patient underwent lung biopsy, and histopathological analysis of the biopsy sample indicated cryptococcosis (Figure 1D).

Pulmonary cryptococcosis is caused by fungi of the genus *Cryptococcus* (*C. neoformans* and C. *gattii*), which are monomorphic encapsulated yeasts that are found worldwide, particularly in soil contaminated with pigeon droppings and decomposing wood. Although infection occurs through the inhalation of airborne infectious particles, pneumonia is relatively uncommon in infected individuals. In fact, after hematogenous dissemination, infection of the central nervous system is more common than is pneumonia^1-4^.

Pulmonary cryptococcosis has a variety of clinical and pathological presentations. It can manifest in immunocompetent and immunocompromised patients, although the latter account for the majority of cases, with a wide variety of radiological abnormalities. The following are the main characteristics to be identified by CT^2-4^: location and distribution; solitary or multiple nodules that can progress to confluence or cavitation; segmental consolidation or infiltrative masses; hilar or mediastinal lymph node enlargement; pleural effusion; reticular or nodular infiltrate; linear opacities; septal thickening; and endobronchial lesions. The diagnosis of pulmonary cryptococcosis is difficult to make, because the organisms often colonize the upper airways and the symptoms are nonspecific, as are the radiological manifestations^4^.

In the diagnosis of pulmonary cryptococcosis, PET/CT plays a complementary role. In approximately 60% of patients, cryptococcal lesions show ^18^F-fluorodeoxyglucose uptake that is greater than is that of the mediastinal blood pool. The SUV, a calculated measure of contrast uptake, is used in order to identify the underlying cause of such lesions, knowledge of their physiological distributions and variants being of fundamental importance for minimizing errors of interpretation^1,5,6^. Typically, low SUVs (≤ 2.5) are associated with benign lesions, whereas high SUVs (> 2.5) are associated with malignant lesions^1,5^. Sharma et al.^1^ demonstrated that the SUVs of cryptococcal lesions range from 0.93 to 11.6.

When PET/CT is used in order to differentiate pulmonary nodules and to discriminate between infection and malignancy, its potential pitfalls should be borne in mind, especially in areas where the prevalence of granulomatous infection is high, as well as in immunocompromised patients^7,8^. Inflammatory and infectious lesions can show elevated metabolic rates and can therefore be misidentified as malignant lesions^5,6,8^, thus posing a diagnostic challenge^6^.

In patients with pulmonary cryptococcosis, there is great variability in the SUVs of cryptococcal lesions. Therefore, the clinical correlation, risk factors for cancer development, and geographic location, together with the PET/CT findings, are fundamental for diagnostic clarification, although it is usually necessary to perform a lung biopsy^1,5,8^.
